# Radiographic prediction of metallic foreign body penetration in the reticulum of cows and buffaloes

**DOI:** 10.14202/vetworld.2018.488-496

**Published:** 2018-04-16

**Authors:** Shanib Mehraj Makhdoomi, Vandana Sangwan, and Ashwani Kumar

**Affiliations:** Department of Veterinary Surgery and Radiology, College of Veterinary Science, Guru Angad Dev Veterinary and Animal Sciences University, Ludhiana - 141 004, Punjab, India

**Keywords:** bovine, cows, cranioventral abdomen, radiograph, reticulum

## Abstract

**Aim:**

This study aimed to evaluate the role of radiography in the standing (right and left) and recumbent (right) lateral positions for the detection and prediction of metallic foreign body penetration in the reticular wall.

**Materials and Methods:**

A total of 41 bovines (23 cows and 18 buffaloes) having at least one sharp metallic foreign body (>1 cm) detected on reticular radiographs were investigated, and their extent of penetration in the reticular wall was confirmed on the left flank laparorumenotomy.

**Results:**

Of total sharp metallic foreign bodies retrieved on rumenotomy, the maximum percent were detected on the right recumbent radiographic view (75.00% in cows and 57.14% in buffaloes) compared to the right standing (54.38% in cows and 40.42% in buffaloes) and left standing (51.06% in cows and 27.08% in buffaloes) radiographic views. The presence of gas pocket or nodule adjoining a foreign body, faintly visible foreign body, foreign body that appeared partially or completely out of the reticulum, and foreign body that appeared parallel, into, or directed toward the diaphragm indicated a high probability in the prediction of penetrating foreign body in the left standing (100%) followed by the right recumbent (85.71% in cattle and 90% in buffaloes) and right standing (94.74% in cattle and 55.56% in buffaloes) radiographic views.

**Conclusion:**

The right recumbent radiographic view is most reliable to detect sharp metallic foreign bodies in bovine. Buffaloes engulf more number of foreign bodies; however, comparatively, the number of completely or partially penetrating foreign bodies is high in cattle. The hypothesized radiographic parameters for the prediction of penetrability of the metallic foreign body were 100% reliable in the left standing radiographic view in both the species.

## Introduction

Ingestion of foreign bodies is a common problem in bovines. The reasons reported for this are incomplete mastication of feed before swallowing, unselective feeding habit, tongue as the prehensile organ, and increased mechanization [1,2]. The ingested foreign bodies may injure or penetrate the reticular wall, and the condition is known by various names such as foreign body syndrome, traumatic reticuloperitonitis (TRP), hardware disease, or sharp foreign body syndrome [3,4]. Non-specific clinical signs of reduced appetite, tympany, and abnormal defecation are described in buffaloes suffering from TRP [5]. Economic losses due to a reduction in milk and meat production, treatment costs, and potential fatalities in TRP affected bovines had driven researchers to go deep in the diagnosis and treatment of this syndrome [3,6].

The metal detectors were earlier used to identify the metal in the reticulum, but they do not distinguish between perforating and non-perforating foreign bodies, whereas radiographs apart from locating the foreign bodies also provide sufficient information concerning the nature and extent of damage caused by the potential foreign bodies [7-9]. The metallic foreign bodies in the reticulum may be classified as potential (such as wires, needle, and nails which are sharp and metallic on radiograph and appear to have the potential to penetrate the reticular wall or the adjoining structures) or non-potential (such as nut bolts, key, chain, coins, rings, anklets, and stones which may be metallic or non-metallic on radiograph and do not appear sharp enough to penetrate the reticular wall) [10]. However, due to inherent radiographic image distortion, certain foreign bodies which do not appear to be potential on radiograph may be potential or penetrating on rumenotomy or vice versa. The various parameters observed on radiographs for the diagnosis of the TRP include the presence or absence of a foreign body, the presence of focal gas shadows or gas fluid interface near the reticulum, shape size, and location of the reticulum [11]. However, peritoneal effusions, reticular abscesses, and diaphragmatic hernias can be better visualized on ultrasonography [12-17]. There is scanty literature on the radiographic prediction of penetrating and non-penetrating foreign bodies in the reticular region in cattle [18-20]; however, no literature is available in buffaloes. Radiography of the reticulum is preferred in standing position in cattle for the diagnosis of TRP to avoid other complications of spreading the infection [21], but standing radiographs, particularly in buffaloes, may have less diagnostic value in reticular affections. As per the author’s knowledge, there is no published literature on the comparative radiographic features of TRP affected reticulum in cattle and buffaloes in various radiographic positions.

Therefore, the present study was carried out to evaluate the role of radiography in the detection and prediction of metallic foreign body penetration in the reticular wall in cows and buffaloes.

## Materials and Methods

### Ethical approval

This clinical study was duly approved by the Institutional Animal Ethics Committee.

### Animals

A total of 41 bovines (23 cross-bred Indian cows [*Bos taurus* and *Bos indicus*] and 18 buffaloes [*Bubalus bubalis*]) suffering from foreign body syndrome and having at least one sharp metallic foreign body (SMFB) in the reticular region in any radiographic view and were subjected to surgical intervention for the removal of foreign body were included in the study.

### Radiographic examinations

All the bovines were subjected to reticular radiography in recumbent (left to right lateral) and standing (left to right and right to left lateral) positions except 10 bovines which were considered risky for radiography in multiple positions due to acute bloat (3 cows) or advanced pregnancy (6 cows) or non-functioning of X-ray machine (one buffalo). The left to right lateral was denoted as “right lateral” and the right to left lateral as “left lateral” throughout the text and figures. Radiography was done using ceiling-mounted movable Siemens 800 mA X-ray machine. The radiographic exposure factors used were 90-113 KVp, 53 mAs, and 90-110 cm as film focus distance. The radiographs were processed using Kodak computerized radiography (CR) system.

The various radiographic parameters recorded in the study were:

1. The number of SMFB’s apparent on various radiographic views in cows and buffaloes.
2. The size of the SMFB seen on various radiographic views in cows and buffaloes. It was measured in centimeter using the inbuilt caliper in the CR system software. SMFB’s distinctly measured to be more than 1cm in length were included in the study [19].
3. The distance of the reticulum from the diaphragm (reticulodiaphragmatic separation) was measured in various radiographic views at the ventral region in all the bovine (Figure-1).

**Figure-1:**
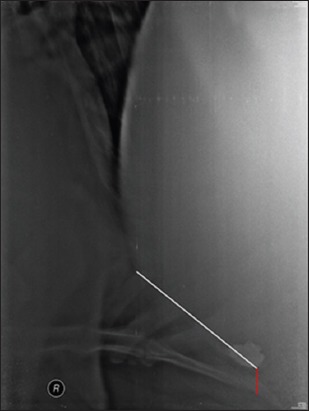
Radiograph showing the length of reticulum (white line) and the distance of reticulum from the diaphragm in ventral region (red line).
4. The length of reticulum was measured from the caudoventral tip of the reticulum to its cranial border at the point of intersection of the costochondral junction (Figure-1).
5. The presence of gas pockets adjoining to the SMFB (Figure-2).

**Figure-2:**
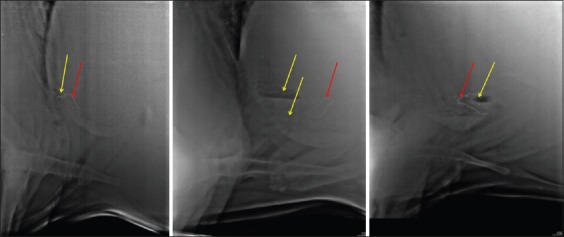
Radiographs showing gas pockets (yellow arrow) around the sharp metallic foreign body (red arrow).
6. The SMFB that appeared partially or completely out of the reticulum (Figure-3).

**Figure-3:**
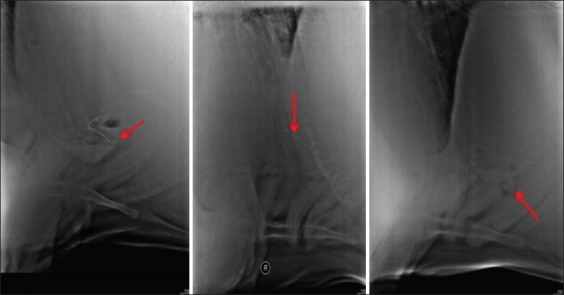
Radiographs showing sharp metallic foreign body (red arrow) partially or completely out of reticulum.
7. The SMFB that appeared faint on the radiograph (Figure-4).

**Figure-4:**
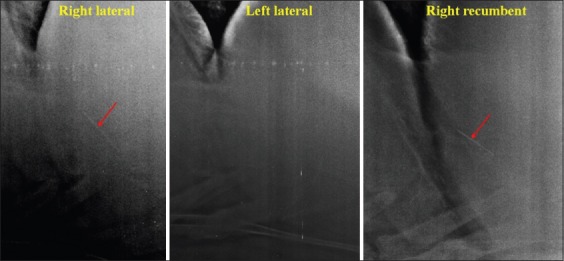
Radiograph showing faintly visible (right standing and right recumbent) or non-visible (left standing) sharp metallic foreign body (red arrow).
8. The SMFB that appeared in a nodule; partially or completely (Figure-5).

**Figure-5:**
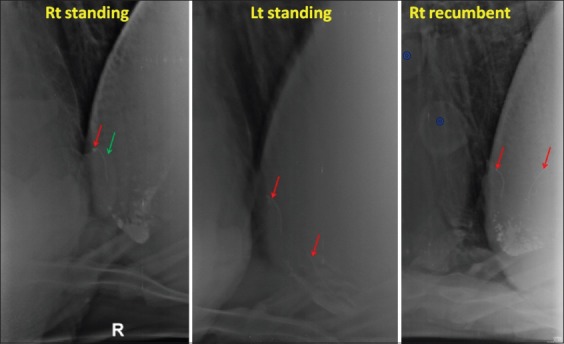
Radiographs showing sharp metallic foreign body (SMFB) (red arrow) in a nodule (green arrow) in the right standing view. Another SMFB is seen in the left standing and right recumbent view. Two soft tissue opacities (hollow blueblue hollow circles, possibly cysts) are also seen in the right recumbent view.
9. The SMFB that appeared parallel to the diaphragm (Figure-6).

**Figure-6:**
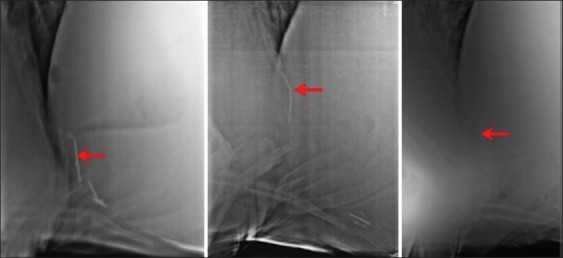
Radiographs are showing sharp metallic foreign body (red arrow) parallel to diaphragm.
10. The SMFB that appeared partially into the diaphragm or directed toward diaphragm (Figure-7).

**Figure-7:**
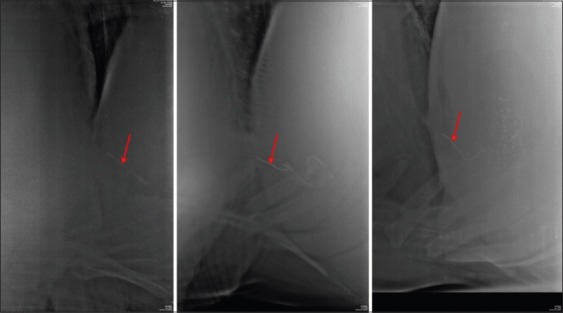
Radiographs are showing sharp metallic foreign body (red arrow) directed toward the diaphragm.
11. The position of the SMFB in the reticulum on the radiograph. The reticulum was divided into six quadrants by three imaginary lines drawn on the lateral radiograph. Two horizontal lines; one from the cupula and the other from the proximal tip of 7^th^ sternebra were drawn. One vertical line was drawn dorsally from the distal tip of 7^th^ sternebra (Figure-8).

**Figure-8:**
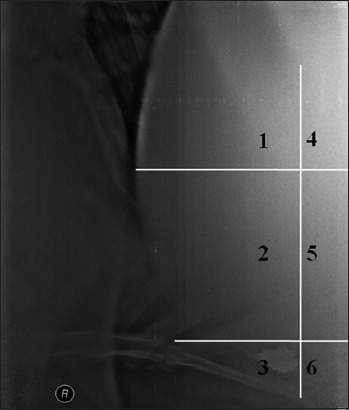
Radiograph showing the six quadrants used to ascertain the position of the sharp metallic foreign body in the reticular region.
12. The angle of the SMFB with the sternum and the diaphragm (Figure-9) was measured and was categorized as <30° or >30° [20].

**Figure-9:**
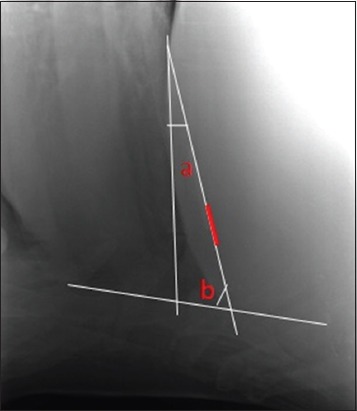
Radiograph showing the measurement of the angle of the sharp metallic foreign body (red line) in reference to the sternum (b) and diaphragm (a).

### Confirmatory diagnosis

All the bovines were subjected to the left flank laparorumenotomy for the retrieval of foreign bodies from the reticulum. The retrieved foreign bodies were matched with those seen on radiographs and were classified as completely penetrating (CP; which were not located free within the reticular lumen, rather were either felt sliding in the reticular wall with the diaphragm or through some nodule, abscess or inflammatory reaction at the suspected site, and a blind stab incision was required on the reticular wall for their retrieval), partial penetrating (PP; when a small portion of foreign body was felt within the reticular lumen while the remaining portion of the foreign body was piercing the wall or the adjoining structures and was retrieved by pulling into the reticular lumen), and the non-penetrating (NP; when the foreign bodies were found lying free within the reticular lumen).

### Statistical analysis

The data generated were subjected to statistical analysis using Microsoft Excel. The mean and the standard deviation of all the numerical parameters were calculated and compared between the cattle and buffaloes and in relation to the extent of penetration of the SMFB’s using Student’s t-test at 1% and 5% level of significance. The subjective data were interpreted on relative percentage basis.

## Results

**Figure-10:**
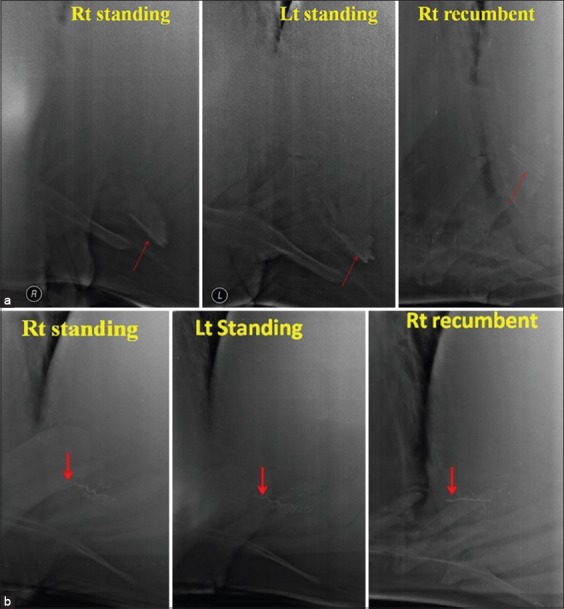
(a) Radiographs showing visibility of sharp metallic foreign body (SMFB) (red arrow) in various positions in Buffalo with one visible SMFB in the right standing, 2 in the left standing, and 4 in the right recumbent view. (b) Radiographs showing visibility of SMFB (red arrow) in various positions in a cow with one SMFB seen partially out of the cranial reticular wall in the right standing and recumbent views but not in the left standing view.

In this study, a total maximum of 58 SMFB’s in 23 cows (average 2.5/cow) and 57 in 18 buffaloes (3.2/buffalo) were retrieved on rumenotomy (Table-1). The maximum percent of foreign bodies were seen on the right recumbent radiographic view (75% in cows and 57.14% in buffaloes) compared to the right standing (54.38% in cows and 40.42% in buffaloes) and left standing (51.06% in cows and 27.08% in buffaloes) (Figure-10a and b). In buffaloes, irrespective of the radiographic view, a maximum of 57.14% SMFB’s could only be detected suggesting that reticular radiography could not be reliably used to rule out the presence of SMFB’s in buffaloes. In contrast, in majority of the cows, at least one SMFB was seen in at least one radiographic view, except in two cases, where no SMFB was seen and instead a bunch of foreign bodies was seen in standing position. In one case, the SMFB was attached to the magnet, while in another case, the foreign body was completely out of reticulum (retrieved on rumenotomy) and the magnet was lying free in the reticulum. In buffaloes, however, there were 4 (30.76%) and 5 (38.46%) cases where no foreign body was visualized in the right standing and left standing radiographic views, respectively.

**Table-1:** Distribution of SMFB’s in various radiographic views in TRP affected cows and buffaloes.

Species	Cows (n=23)			Buffaloes (n=18)		
		
Radiographic view	Right lateral Recumbent	Right lateral Standing	Left lateral Standing	Right lateral Recumbent	Right lateral Standing	Left lateral Standing
Number of bovine radiographed	14	22	21	17	13	13
Number of SMFB’s seen	24	31+2 bunches	24+2 bunches	32+1 bunch	19	13+1 bunch
Number of SMFB’s retrieved	32	57	47	56	47	48
Overall number of SMFB’s recovered in rumenotomy	58			57		
CP/PP/NP retrieved out of respective views taken	6/12/14	10/26/21	11/17/18	4/11/41	3/10/34	3/10/35
Overall CP/PP/NP	11/26/21=58			4/12/41=57		
retrieved	2.75:6.5:5.25			1:3:10.25		
Average SMFB’s per bovine	2.5			3.2		
% positive predictive value	24/32=75%	31/57=54.38%	24/47=51.06%	32/56=57.14%	19/47=40.42%	13/48=27.08%

SMFB=Sharp metallic foreign body, TRP=Traumatic reticuloperitonitis, CP=Completely penetrating, PP=Partial penetrating, NP=Non-penetrating

The average numbers of SMFB’s were more in buffaloes (3.2 per buffalo) compared to cows (2.5 per cow), but the number of CP foreign bodies was more in cows (n=11, 18.97%) compared to buffaloes (n=4, 7.02%) signifying the fact that the removal of foreign bodies should be done early in cattle to prevent its perforation into the reticular wall. The ratio of foreign bodies retrieved in cows and buffaloes based on penetrability was 2.75:1 (CP), 6.5:3 (PP), and 5.25:10.25 (NP), respectively.

The length of SMFB retrieved on rumenotomy varied from 2 to 10 cm in cattle and 1.9 to 15.5 cm in buffaloes. The length of the SMFB was found to be important in assessing its penetrating status, as all the SMFBs more than 5.5 cm were found to be penetrating (PP or CP), in both the species.

**Figure-11:**
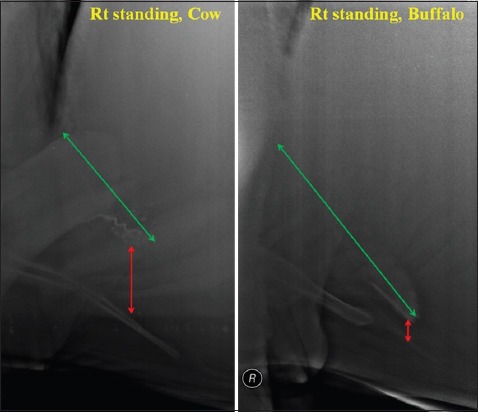
Radiograph showing comparative lifting (red arrow) and shortening (green arrow) of reticulum in traumatic reticuloperitonitis cow and buffalo.

The mean±standard deviation distance of reticulodiaphragmatic separation at ventral region (lifted reticulum) was significantly (p<0.05) more in cattle having PP (3.98±2.16 cm) and CP (3.36±1.79 cm) SMFB’s when compared to NP (1.4±0.81 cm) in the right standing radiographic position. Similarly, in the left standing position, the distance was significantly (p<0.05) more in cattle having PP or CP SMFB’s (3.81±2.16 cm) compared to NP (1.66±0.81 cm) SMFB’s. The comparison in the right recumbent radiographic view was not possible due to a single value for NP SMFB. While in buffaloes, the ventral distance was found to be significantly (p<0.05) more in PP (2.07±0.89 cm) SMFB’s when compared to CP (1.12±0.37 cm) in the right lateral standing view only. When compared in between the species, the distance was found to be significantly (p=0.05 in recumbent and p=0.0048 in the right standing) more in cattle (irrespective of penetrability) (4.58±2.1 cm in recumbent and 3.31±1.99 cm in the right standing) compared to buffalo (2.97±2.09 cm in recumbent and 1.73±0.82 cm in the right standing) (Figure-11).

In this study, no significant difference was found between the reticular lengths of completely and/or PP and NP SMFB’s (parameter 7) in both the species in any of the radiographic views. However, when compared between the species, a statistically significant (p=0.05) shortening of reticular length was found in cows (15.31±5.22 cm) having SMFB’s (irrespective of its penetrating status) in the right lateral standing radiographs compared to buffaloes (21.07±4.99 cm) (Figure-11). The reticular length was also significantly (p=0.026) short in cows (14.41±6.14 cm) having to penetrate SMFB’s (irrespective of PP or CP) when compared to that in buffaloes (20.37±5.55 cm) in the right lateral standing view.

Based on findings in Table-2, it was concluded that the presence of parameter 5-10 of materials and methods suggested high probability for the prediction of penetration in the left standing radiographic view (100% in both the species), followed by the right recumbent (cows 85.71% and buffaloes 90%), and right standing lateral view (94.74% in cows and 55.56% buffalo). Also that if an SMFB was seen either in a nodule or parallel to the diaphragm or directed toward diaphragm, irrespective of the species, and radiographic view had 100% true predictive value that it is PP or CP in the reticular wall.

**Table-2:** Distribution of SMFB’s in various radiographic views based on criteria’s for penetrability in TRP affected cows and buffaloes.

Parameters	Cows (n=23)			Buffaloes (n=18)			Positive percentage predictive value
	
	Right recumbent	Right lateral	Left lateral	Right recumbent	Right lateral	Left lateral	
SMFB seen partially or completely out of reticulum	2 guessed 1 true (PP) 1 false (NP)	7 guessed 6 true (5 CP-1 PP) 1 false (1 NP)	1 guessed 1 true (PP)	2 guessed 2 true (1 CP-1 PP)	2 guessed 1 true (1 CP) 1 false (1 NP)	2 guessed 2 true (1 CP-1 PP)	13/16=81.25%
SMFB seen with gas pockets	3 guessed 3 true (3 CP)	1 guessed 1 true (1 PP)	2 guessed 2 True (2 CP)	2 guessed 1 true (1 CP), 1 false (1 NP, reticular abscess)	None	None	7/8=87.5%
SMFB faintly seen	None	2 guessed 2 true (1 CP-1 PP)	3 guessed 3 true (3 CP)	1 guessed 1 true (1 CP)	3 guessed 3 false (3 NP in one animal)	None	6/9=66.67
SMFB is seen with nodule	None	2 guessed 2 true (1 CP1PP)	None	None	None	1 guessed 1 true (1 CP)	3/3=100%
SMFB seen parallel to diaphragm in the 2^nd^ Quadrant (Q)	1 guessed 1 true (1 CP)	4 guessed 4 true (2 CP-2 PP)	2 guessed 2 true (2 CP)	1 guessed 1 true (1 PP)	None	None	8/8=100%
SMFB seen in the diaphragm or directed toward the diaphragm	1 guessed 1 true (1 PP)	3 guessed 3 true (2 CP-1 PP)	1 guessed 1 true (1 CP)	4 guessed 4 true (1 CP-3 PP)	4 guessed 4 true (1 CP-3 PP)	5 guessed 5 true (5 PP)	19/19=100%
Percentage positive predictive value	6/7=85.71%	18/19=94.74%	10/10=100%	9/10=90%	5/9=55.56%	8/8=100%	-

SMFB=Sharp metallic foreign body, TRP=Traumatic reticuloperitonitis, CP=Completely penetrating, PP=Partial penetrating, NP=Nonpenetrating

On the basis of parameter 11, (Figure-8), in cows, the maximum percent of the SMFB’s were seen in the 2^nd^ Quadrant, irrespective of the radiographic view, or penetrating status of foreign body; however, in buffaloes, the maximum percent of SMFB’s were seen in the 2^nd^ Quadrant, irrespective of the penetrating status in the right recumbent and left standing radiographs only. In cows, it was recorded that the SMFB’s seen in the 1^st^, 5^th^, or 6^th^ Quadrant, irrespective of radiographic view, were 100% suggesting of penetration (PP or CP) suggesting that a foreign body lying on the reticular floor that is 6^th^ Quadrant can also be penetrating. In contrast, in buffaloes, the majority of the SMFB’s (90.47%; 19 out of 21) seen in the 1^st^, 5^th^, and 6^th^ Quadrant in buffaloes were NP.

Based on the data of the angle of the SMFB with the sternum and the diaphragm in cows and buffaloes, the hypothesized criterion of angle of SMFB <30° or >30°with the diaphragm or the sternum for the prediction of penetration of SMFB was not found reliable in various radiographic views in both the species.

## Discussion

In the present study, the average numbers of foreign bodies present in the reticulum were more in buffaloes, but the CP foreign bodies were more in cattle [4]. The center of radiographic exposure was kept at 18-20 cm from the xiphoid at the 6^th^ intercostal space or the 7^th^ rib [19]; however, Braun *et al*. [22] made the center of the radiographic beam for radiography of the reticular region at the 8^th^ rib in cattle. This centering at the 8^th^ rib may be better for the complete visualization of the reticulum, but the part cranial to or at the diaphragm is compromised which otherwise may be required for the diagnosis of reticulodiaphragmatic hernia [16] or to detect an SMFB lying at the diaphragm. Multiple radiographic views were done in this study, to rule out all the possibilities to detect a foreign body on a radiograph. Although previous studies recommend avoiding recumbent radiography in cases of TRP as it was stressful to the bovine, and there were chances of spreading the infection which otherwise might be encapsulated [18,19]. However, in the present study, standing radiography was not found diagnostic in buffaloes due to their heavy humeral musculature which limits stretching the limb forward during radiographic exposure in standing position.

The left standing radiographic view was found to be least sensitive to the detection of metallic foreign bodies in both the species when compared to the right standing view; however, the right recumbent radiograph was most sensitive. However, the left standing radiographic view was more sensitive in predicting the penetrating status of a metallic foreign body based on the parameters taken in this study when compared to the right standing.

The significant increase in the reticulodiaphragmatic separation on the ventral aspect suggests that the cattle are prone to developing localized peritonitis in cases of SMFB’s (irrespective of its extent of penetrability) compared to buffaloes, which lifts the reticulum and thus increase this distance on the radiograph. The increased reticulodiaphragmatic separation at cranial and ven­tral positions not correlate with a specific disease process (such as hepatic, TRP, and reticular abscess) in cattle [19].

In cattle, the reticular length has been reported to vary, non-significantly, in TRP and significantly in vagal indigestion compared to various reticular conditions [19]. Similar findings were found in the present study for individual species. However, when compared between the species, a significant shortening of the reticular length was found in cows suffering from TRP (irrespective of the extent of penetrability of SMFB’s) compared to buffaloes in various radiographic views which may be suggestive of greater sensitivity to pain in cows.

The embedded SMFB’s in the fibrous tissue, a nodule or reticular mucosa with inflammatory reaction, may appear faint on standing radiograph of the reticular region. The presence of gas lucencies and nodules had been reported to be pathological radiographic features in TRP affected cattle [20], which corroborate findings of this study.

The presence of SMFB, off the reticular floor, may be considered penetrating and those situated flat on the floor of the reticulum or attached to magnet were not considered abnormal or penetrating [18,20]. The reticulum in the present study was lifted in TRP affected cows (irrespective of the extent of penetrability of the SMFB’s), and the 3^rd^ and the 6^th^ Quadrant were usually devoid of the reticular floor. The floor of the reticulum was seen in the 2^nd^ or the 5^th^ Quadrant in the TRP affected cows. It may also be the reason for less number of SMFB’s in the 3^rd^ and 6^th^ Quadrant in cows (n=7) compared to buffaloes (n=22). However, then, the maximum numbers of the foreign bodies were also seen in the 2^nd^ Quadrant in both the species. However, still, in a few cases, the foreign bodies lying flat on the reticular floor were found to be penetrating.

Braun *et al*. [20] reported that a foreign body which had an angle of >30° to the floor is considered penetrating, but this criterion was not found reliable in the present study, though the authors measured the angle both with the sternum and the diaphragm.

## Conclusions

From the present radiographic study, the following conclusions are drawn:

1. To detect an SMFB in bovine, the right recumbent radiographic view is the most, and the left standing is the least sensitive, but also that the right standing view in cows is sufficient to detect at least one SMFB.
2. Non-visualization of SMFB does not rule out TRP in buffaloes even in the right recumbent radiographic view.
3. Buffaloes engulf more number of SMFB’s, but the number of CP or PP SMFB’s is more in cows.
4. SMFB seen off the reticular floor or surrounded by gas pockets or in a nodule or appeared faint or partially/completely out of reticulum or seen parallel, into, or directed toward the diaphragm were found reliable indicators in predicting the penetrating status of the SMFB’s in bovines.

## Authors’ Contributions

SMM, as MVSc Scholar, designed this clinical study under the guidance of VS and AK. SMM took radiographic parameters and guided in the positioning and exposure of the radiographs along with the assistance in performing surgery. VS and AK performed surgeries along with the radiographic interpretation of the data. SMM collected data, analyzed and prepared the draft of the manuscript. VS and AK read, revised, and approved the final manuscript. All authors read and approved the final manuscript.
